# From pitch to palsy: A rare case of acute brachial plexopathy following medial clavicle fracture in women's rugby^☆^

**DOI:** 10.1016/j.tcr.2026.101333

**Published:** 2026-04-29

**Authors:** Imad Mirza, P.J. O'Donoghue, Nicholas Stratford, Emma Carr, Roisin Dolan, Kieran O'Shea

**Affiliations:** a Department of Trauma & Orthopaedic Surgery, St. Vincent's University Hospital, Dublin, Ireland; b Department of Plastic & Reconstructive Surgery, St. Vincent's University Hospital, Dublin, Ireland; c Department of Hand Therapy, St. Vincent's University Hospital, Dublin, Ireland; d UCD School of Medicine & Medical Sciences, University College Dublin, Ireland

**Keywords:** Clavicle fractures, Brachial plexus, Peripheral nerve injuries, Sports injuries, Neuroma

## Abstract

**Background:**

Medial third clavicle fractures represent less than 5% of all clavicle fractures and are rarely associated with acute brachial plexus injury. Early recognition is critical, as timely surgical intervention may influence neurological recovery.

**Case:**

A 17-year-old elite rugby player sustained a closed, displaced medial third clavicle fracture following a high-energy tackle, confirmed on radiographs. Within 48 h, she developed profound C5–C6 sensorimotor deficits. CT angiography excluded vascular injury. MRI demonstrated focal T2 hyperintensity and enlargement of the upper trunk consistent with nerve oedema and axonal injury. The posteriorly displaced medial clavicle fragment compressed the brachial plexus, the sharp edges raised concern for a focal injury. A dual-level injury involving traction at the root and focal injury at the trunk could not be excluded. Nerve conduction studies revealed conduction block without axonal disruption. At two weeks, she underwent open reduction, internal fixation, and brachial plexus exploration. The C5–C6 roots were in continuity, and the upper trunk was contused; external neurolysis was performed. Sensory symptoms resolved by week 3; motor recovery began at week 4; complete functional recovery and return-to-play was achieved at 6-month follow-up.

**Conclusion:**

Early imaging and decompression are essential to optimise recovery in medial clavicle fractures with acute brachial plexopathy.

## Highlights

• Brachial plexus injury represents a rare but significant complication of clavicle fractures
• Profound neurological deficits mandate thorough neurovascular examination and early MRI.
• Root continuity does not exclude clinically significant compression.
• Safe decompression can be achieved through open reduction and internal fixation of the clavicular fracture combined with targeted supraclavicular plexus exploration
• Early safe decompression should facilitate optimal conditions for functional recovery.

## Background

Clavicle fractures account for 2.6–4% of all fractures, yet medial third fractures comprise only 2–5% of these injuries [1], [2]. First-line treatment is usually nonoperative, with sling immobilisation, as most fractures unite uneventfully. Operative fixation is reserved for open fractures, skin tenting, displacement ≥2 cm, comminution, or neurovascular compromise.

Acute brachial plexus injury (BPI) accompanying clavicle fracture is distinctly uncommon. Among reported series, fewer than 1% of clavicle fractures present with immediate plexopathy [3]. Published cases more often describe plexopathy appearing days to months later, secondary to hypertrophic callus, scarring, or iatrogenic injury after fixation [4], [5].

We report an elite female rugby player who presented with a displaced medial third clavicle fracture and simultaneous upper trunk brachial plexus palsy, following a high-energy tackle, who recovered rapidly following early open fracture reduction and internal fixation with concomitant brachial plexus exploration, decompression and neurolysis.

## Case presentation

A 17-year-old elite female rugby back-row player sustained a head-on collision during a game. The opposing player impacted her left shoulder with downward traction. She immediately reported severe shoulder girdle pain and presented to the emergency department.

**Fig. 1:**
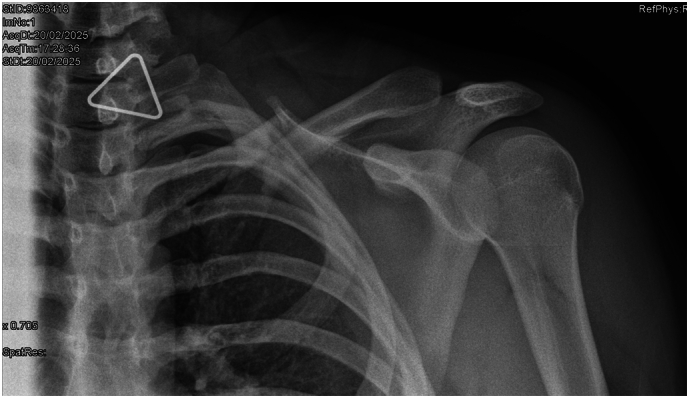
Initial radiograph of left clavicle showing mid shaft clavicle fracture with displacement.

Initial assessment confirmed a displaced medial third fracture of the left clavicle (Fig. 1). Within 48 h, she slowly developed profound sensorimotor deficits affecting the ipsilateral upper limb. Clinical examination revealed asymmetric posturing and an adducted, internally rotated resting posture of the affected limb. Motor testing demonstrated complete paralysis in C5–C6 myotomes, with global palsy of shoulder abduction and external rotation (deltoid, supraspinatus, infraspinatus all MRC Grade 1), scapulothoracic weakness (serratus anterior and rhomboids MRC Grade 1) as well as elbow flexion and forearm supination weakness (biceps, brachialis, brachioradialis MRC Grade 1). Partial weakness was noted in wrist extension (radial-innervated C6 component MRC Grade 3), while more distal radial, median, and ulnar motor sensorimotor functions were preserved. Sensory examination showed anaesthesia in the C5–C6 dermatomes, involving the lateral aspect of the shoulder, arm, and forearm (S0). Sensation in the hand (C7–T1 distribution) was intact. Horner's syndrome was absent, and a chest radiograph revealed no evidence of raised hemidiaphragm.

**Fig. 2:**
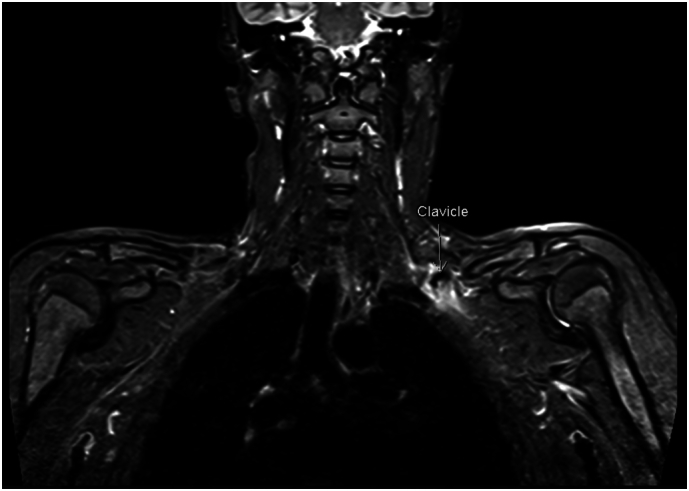
MRI Brachial plexus showing thickened and oedematous brachial plexus, at the site of clavicle fracture impingement.

CT angiography excluded vascular injury, and MRI c-spine was normal. MRI left brachial plexus demonstrated focal T2 hyperintensity and enlargement of the upper trunk consistent with nerve oedema and axonal injury (Fig. 2). The brachial plexus was compressed by the posteriorly displaced medial clavicle fragment, the sharp edges of which raised concern for a focal sharp injury. Given the mechanism, a dual-level injury involving stretch at the root level and a focal injury at the trunk level could not be excluded. Nerve conduction studies, performed at day 10, already confirmed conduction block in the deltoid, biceps, infraspinatus, and triceps without spontaneous activity, indicating preserved axonal continuity.

**Fig. 3:**
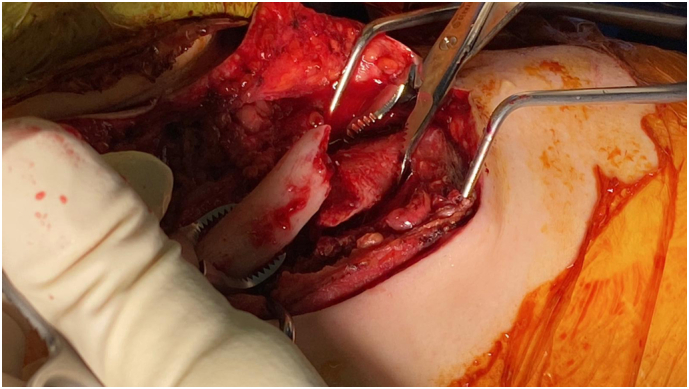
Intraoperative clinical photo highlighting the clavicle fracture with displacement.

**Fig. 4:**
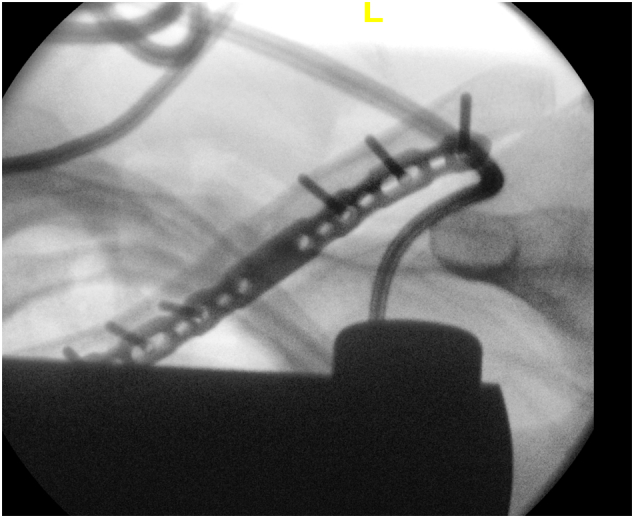
Intraoperative Screening image showing satisfactory application of clavicle plate.

**Fig. 5:**
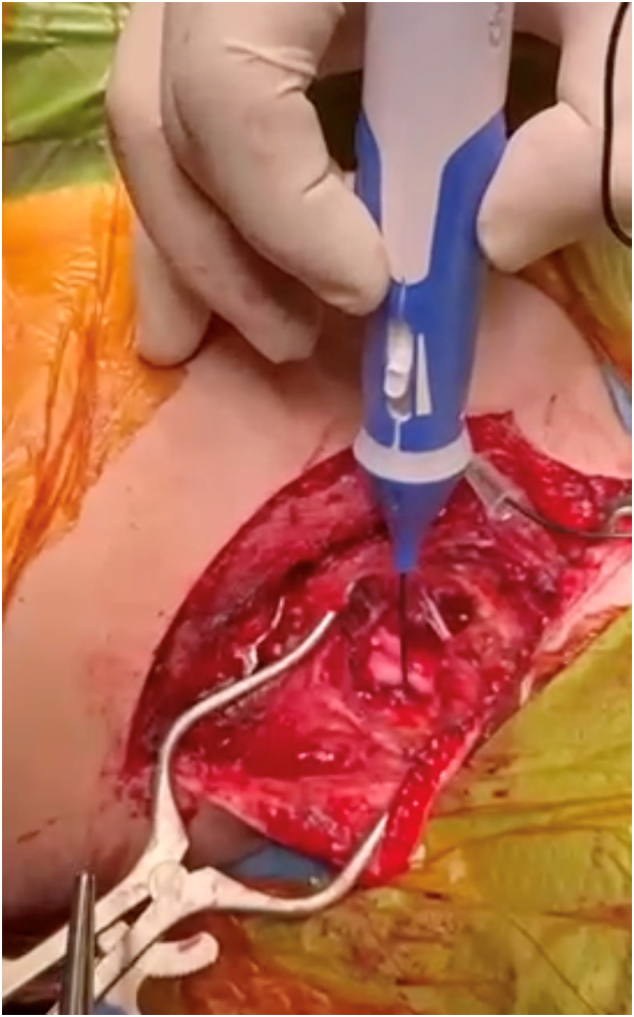
Intraoperative clinical photograph showing nerve stimulation of thickened and oedematous C5 nerve root.

Two weeks post-injury, the patient underwent open reduction and plate fixation of the clavicle with concurrent supraclavicular plexus exploration (Fig. 3, Fig. 4). Intra-operatively, the phrenic and long thoracic nerves responded to stimulation. Early fusiform swelling and contusion of the affected upper trunk segment was noted and elicited no response. C5 and C6 roots were identified intact. External neurolysis of the upper trunk was performed (Fig. 5).

Postoperatively, hypersensitivity resolved by week 3; biceps contraction returned at week 4; by week 10, she regained full passive and near-full active shoulder motion with full sensory recovery (Fig. 4).

## Discussion

Acute brachial plexopathy occurs in less than 1% of clavicle fractures, yet, when present, it demands early recognition and targeted intervention [6]. Medial third fractures represent only 2–5% of clavicle fractures, making the concurrence with brachial plexopathy exceptionally rare [7]. The mechanisms involved include high-velocity traction with contralateral neck displacement risking root avulsion, and direct compression of the plexus by fracture fragments, haematoma, or hypertrophic callus. Our patient likely experienced a dual mechanism involving downward traction, causing stretch of C5 and C6 nerve roots and upper trunk extrinsic compression from the clavicle fracture, as demonstrated on MRI.

MRI was a helpful diagnostic adjunct. The lack of pseudomeningoceles supported the hypothesis that C5 and C6 were likely to be intact, confirming mechanical extrinsic compression from the fracture fragments. Electrodiagnostics supported a Sunderland grade II–III injury, with preserved axonal continuity but absent motor unit potentials in the C5 and C6 distributions [8]. Given the progression of upper limb weakness and evidence of upper trunk compression on MRI, early surgical intervention was undertaken. Intra-operative findings confirmed contusion and early fusiform swelling of the upper trunk with intact C5 and C6 nerve roots. External neurolysis was performed, and the patient demonstrated rapid recovery consistent with focal demyelination.

While some advocate conservative management in compressive plexopathy with intact roots, delayed exploration has been associated with irreversible intraneural fibrosis and poorer outcomes [9], [10]. In athletes and high-functioning individuals, early decompression may mitigate Wallerian degeneration and facilitate timely remyelination within an early regenerative window [11], [12], [13].

## Conclusion

**Fig. 6:**
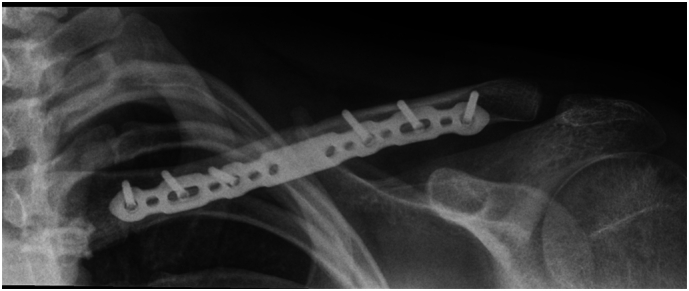
Radiograph of left clavicle at 3 months post operation, showing satisfactory alignment and position of left clavicle.

Medial third clavicle fractures are rare, and their association with acute upper trunk brachial plexopathy is rarer still. Profound C5–C6 deficits mandate thorough neurovascular examination and early MRI, recognising that root continuity does not exclude clinically significant compression. Early open reduction and internal fixation of the clavicular fracture combined with targeted supraclavicular plexus exploration provides safe decompression and should facilitate optimal conditions for functional recovery (Fig. 5, Fig. 6).

## CRediT authorship contribution statement

**Imad Mirza:** Writing – original draft, Data curation. **P.J. O'Donoghue:** Writing – original draft, Methodology. **Nicholas Stratford:** Investigation, Data curation. **Emma Carr:** Investigation. **Roisin Dolan:** Writing – review & editing, Supervision, Conceptualization. **Kieran O'Shea:** Supervision.

## Consent

Written informed consent was obtained from the patient and her guardian for publication of this case report and the accompanying images.

## Declaration of competing interest

The authors declare that they have no known competing financial interests or personal relationships that could have appeared to influence the work reported in this paper.

## Appendix A Supplementary data

Supplementary Video S1 Intraoperative nerve stimulation of the upper trunk of the brachial plexus

Supplementary Video S2 Video taken of the patient at 6 months post-op showing significant functional recovery

Supplementary Video S3 Video taken of the patient at 6 months post-op showing significant functional recovery
